# Computational and Experimental Evaluation of a Flow-Conditioning Anastomotic Device for Arteriovenous Fistula Maturation

**DOI:** 10.3390/bioengineering13091089

**Published:** 2026-09-19

**Authors:** Keith Saum, Begoña Campos, Diego Celdran Bonafonte, Liran Oren, Albert Phillip Owens, Prabir Roy-Chaudhury

**Affiliations:** 1 Division of Nephrology and Hypertension, College of Medicine, University of Cincinnati, Cincinnati, OH 45221, USA; 2 Division of Nephrology, Department of Internal Medicine, University of Michigan, 5313 Brehm Center, 1000 Wall St, Ann Arbor, MI 48105, USA; 3 Division of Nephrology, College of Medicine, University of Arizona, Tucson, AZ 85714, USA; 4 University Animal Care Department, University of Arizona, Tucson, AZ 85714, USA; 5 Department of Otolaryngology–Head and Neck Surgery, College of Medicine, University of Cincinnati, Cincinnati, OH 45221, USA; 6 Division of Cardiovascular Health and Disease, College of Medicine, University of Cincinnati, Cincinnati, OH 45221, USA; 7 Division of Nephrology and Hypertension, Department of Medicine, University of North Carolina, Chapel Hill, NC 27599, USA

**Keywords:** hemodialysis, vascular access, endovascular implant, computational fluid dynamics, wall shear stress, disturbed flow, particle image velocimetry

## Abstract

The arteriovenous fistula (AVF) is the preferred method of vascular access for hemodialysis; however, 30–50% of AVFs undergo primary maturation failure and are unsuitable for clinical use. As disturbed hemodynamics initiate endothelial injury and intimal hyperplasia, we designed an endovascular flow-conditioning anastomotic device (FCAD) to directly improve AVF hemodynamics and protect the anastomotic region. Using computational fluid dynamics, we characterized the flow field and wall shear stress (WSS) profiles in idealized AVF models with and without the FCAD. Incorporation of the FCAD into a brachiocephalic AVF model reduced regions of oscillatory WSS and generated a symmetrical flow profile in the draining vein compared to a reference AVF. Parametric studies identified an FCAD geometry with a tab angle, height, and aspect ratio of 30°, 0.1 diameters, and 1.0 that increased time-averaged WSS along the inner venous wall and achieved a physiological WSS (1–3 Pa) without inducing regions of oscillatory flow throughout the cardiac cycle. Similar findings were observed with an in vitro model using particle image velocimetry. This study demonstrates the feasibility of the FCAD to normalize venous flow and WSS while imposing minimal resistance to blood flow. Restoring physiological WSS levels on the venous wall may help preserve endothelial function and improve AVF maturation.

## 1. Introduction

The arteriovenous fistula (AVF) is the preferred form of vascular access for hemodialysis; however, 30% to 50% of AVFs fail to mature and develop an adequate blood flow or luminal diameter to support hemodialysis [1]. AVF maturation failure results from intimal hyperplasia (IH) and a lack of outward remodeling, leading to progressive occlusion of the venous anastomosis and draining vein [2]. While the exact mechanisms underlying AVF maturation failure remain unclear, considerable evidence suggests that disturbed hemodynamics within an AVF play an important role in IH formation. Specifically, regions of oscillatory flow with low wall shear stress (WSS) appear to correlate with sites vulnerable to IH formation [3]. These disturbed hemodynamics may alter local endothelial cell function and initiate the development of IH [4]. In light of this understanding, hemodialysis could benefit from a novel vascular access technology that restores physiological hemodynamics within the AVF and draining vein.

Several studies have sought to minimize WSS abnormalities and adverse venous remodeling by identifying the optimal anatomical configuration for an AVF [5,6]. However, as others have concluded, it is very difficult to identify a configuration that abolishes all areas of disturbed WSS [3]. Computational analyses of mature, patent fistulae show a time-averaged WSS of approximately 1 Pa in the outflow vein at locations remote from the anastomosis, with values well above this persisting within the anastomosis itself [7]. Prospective duplex ultrasound data from the Hemodialysis Fistula Maturation Study cohort similarly report median venous WSS falling from approximately 3 Pa on postoperative day 1 to approximately 2 Pa by week 6 as the vein dilates [8]. This physiological WSS range of 1–3 Pa must be reached within a short distance, since the sites most vulnerable to intimal hyperplasia lie within the first few centimeters of the anastomosis. Therefore, a more attainable goal may be to combine a reasonable AVF configuration with a way to condition venous flow directly.

Flow conditioning is a method used to alter the flow profile of a fluid downstream of a disturbance or obstruction [9]. Flow-conditioning devices consist of mechanical damping elements inserted into the lumen of a conduit, such as honeycomb vanes, perforated plates, tabs, and tube bundles. In particular, tab-type flow conditioners have proven most effective for particulate mixtures due to the tab’s tapered design, which minimizes fouling and pressure losses [9,10]. In addition, they are the only geometry that can be retrofitted into elbows, similar to an AVF configuration. For these reasons, we hypothesized that incorporating tab-type flow-conditioning elements into an endovascular prosthesis at the AVF anastomosis would reduce regions of low, oscillatory WSS along the outflow vein of a fistula without imposing significant resistance to AVF blood flow.

**Figure 1:**
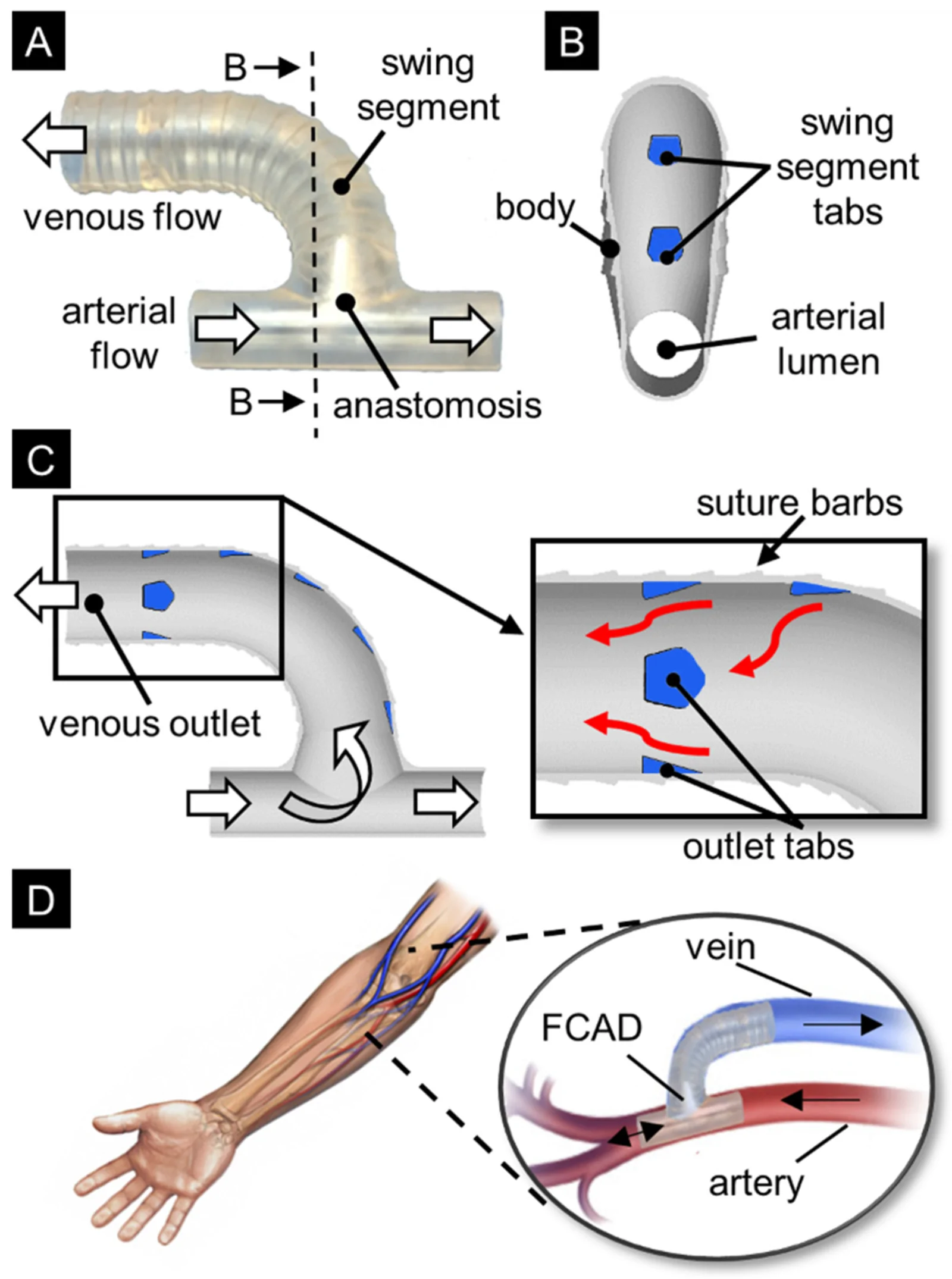
FCAD design and operation principle. (**A**) Prototype FCAD featuring a 90° anastomotic venous swing segment. Arrows indicate the direction of blood flow through the device. (**B**) Transverse section through the anastomosis showing flow-conditioning tabs highlighted in blue along the venous swing segment. (**C**) Longitudinal section through the FCAD lumen showing a second set of flow-conditioning tabs at the venous outlet. Red arrows indicate the direction of blood flow around the tabs. (**D**) Endovascular implantation during AVF creation. Black arrows indicate the main direction of blood flow.

To address this problem, we have developed a novel flow-conditioning anastomotic device (FCAD) to improve AVF maturation by normalizing venous flow and protecting the anastomotic region [11]. The prosthesis consists of arterial and venous lumens coupled to mimic an end-to-side anastomosis (Figure 1A), with flow-conditioning tabs along the outer curve and outlet of the venous swing segment (Figure 1B,C). Rather than relying on anatomy, these tabs act directly on the flow, recovering a symmetric velocity profile at the venous outlet over a shorter length than a native AVF requires. Clinically, the arterial and venous segments would be implanted into their respective lumens before the anastomosis is created (Figure 1D), standardizing the AVF configuration while preserving access for thrombolysis and angioplasty should clot formation occur. As a first step toward developing this technology, this study aimed to characterize the FCAD’s hemodynamic effect relative to an idealized AVF under matched flow conditions, determine how tab geometry governs the venous flow field and flow resistance, and experimentally validate the computed flow profiles by particle image velocimetry.

## 2. Materials and Methods

### 2.1. Idealized AVF Models with the FCAD

**Figure 2:**
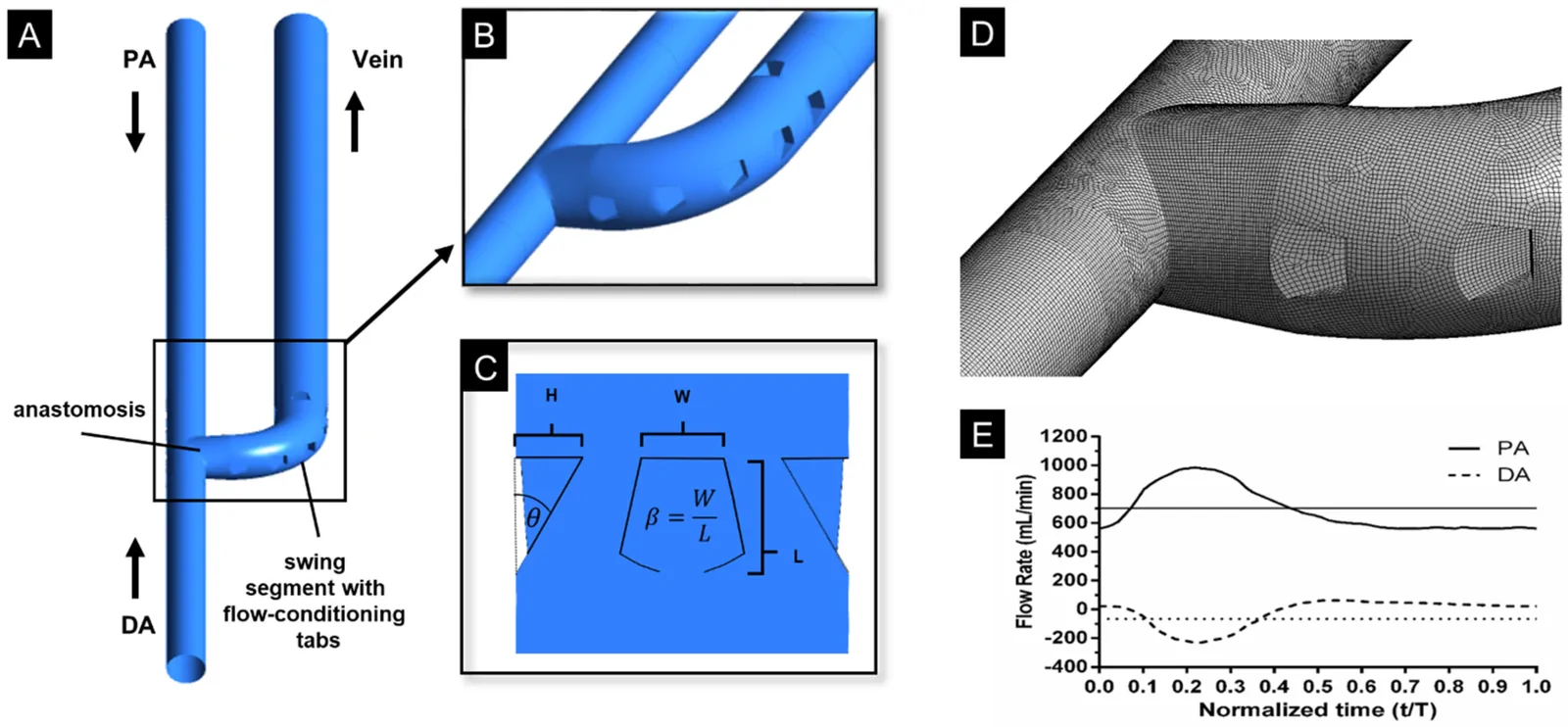
Idealized AVF model with FCAD geometry for CFD simulations. (**A**) 3D surface of the idealized AVF. Black arrows indicate the direction of blood flow in the proximal artery (PA), distal artery (DA), and venous outlet. (**B**) Detailed view of the flow conditioning tabs along the venous swing segment and outlet. (**C**) Detailed view of the tab dimensions at the venous outlet used in the parametric analysis including the tab angle (θ), height (H), and aspect ratio (β). (**D**) Surface mesh at the anastomotic region. (**E**) Volumetric flow rate waveforms of a brachiocephalic AVF obtained from He et al [12]. Continuous and dashed curves represent blood flow rate in PA and DA, respectively. Blood flow in the DA changes direction during the cardiac cycle; negative flow is antegrade (towards the hand) and positive flow is retrograde (towards the anastomosis). Horizontal lines indicate the time-averaged blood flow rate over the entire cardiac cycle, 705 mL/min for PA and −22 mL/min for DA, respectively.

Idealized models of an end-to-side brachiocephalic AVF with and without the FCAD implant were generated using SolidWorks 2015 (Dassault Systèmes, Velizy-Villacoublay, France). We generalized the vessel shape, acknowledging that each patient has a unique vessel size and shape. The artery and vein were modeled as rigid circular cylinders with vessel diameters of 4.0 and 6.0 mm, respectively, with an anastomotic angle of 90°. The length of the proximal artery (PA), distal artery (DA), and vein was 12 times the vessel diameter in order to have sufficient hydraulic length to establish fullydeveloped flow and to characterize the flow field in the vein downstream of the anastomosis (Figure 2A). The swing segment of the venous branch was generated with a radius of curvature equal to twice the venous diameter, and the juxta-anastomotic region of the vein was tapered over a length of two diameters to ensure a smooth transition between the arterial and venous sections.

For the FCAD implant models, we generated two arrays of four flow-conditioning tabs along the swing segment of the vein and the outlet of the venous elbow (Figure 2B). To characterize the impact of the tab geometry on the venous flow field, FCAD implant models were generated with four tab angles (15°, 30°, 45°, and 60°), four tab heights normalized to the venous diameter (0.1D, 0.2D, 0.3D, and 0.4D), and four tab aspect ratios (0.75, 1.0, 1.25, and 1.5) as shown in Figure 2C. Table 1 shows the matrix of FCAD models considered in the parametric analysis. We held the tab number and axial spacing fixed and selected them based on the flow-conditioning literature, the location of flow separation predicted in the reference AVF, and constraints on hemodynamic resistance.

The fluid volume of each model was discretized with a structured hexahedral mesh generated in ICEM 16.0 (ANSYS, Inc., Canonsburg, PA, USA). We generated a 5-cell boundary layer with a growth rate of 1.2 at the walls to accurately capture changes in WSS. We generated three meshes of increasing element density for a single model, with and without the FCAD geometry, to estimate discretization error using the grid convergence index (GCI) method [13]. We performed steady-state computational fluid dynamics (CFD) simulations at the venous flow rate at peak systole for each mesh set and used them to estimate mean velocity and mean WSS in the flow domain. Supplemental Table S1 presents the grid convergence results. Velocity and WSS fluctuations due to poor spatial resolution were minimized to less than 5% when the mesh size exceeded 5.4 × 10^6^ and 7.0 × 10^6^ elements in the AVF and FCAD models, respectively. Therefore, we generated similar meshes (~7.0 × 10^6^) for all remaining FCAD models.

### 2.2. CFD Simulations of Blood Flow

Idealized AVF models were imported into the commercial CFD solver ANSYS Fluent 16.0 (Fluent, Inc., Lebanon, NH, USA) and used to simulate pulsatile blood flow in the vessels. The transient Navier–Stokes equations were solved using a laminar model with a pressure-implicit with splitting of operators (PISO) algorithm for the pressure–velocity coupling. As boundary conditions, we prescribed volumetric flow waveforms at the PA and DA inlets (Figure 2E), obtained from He et al. using 2D cine PC MRI of an end-to-side brachiocephalic AVF 5 months after creation [12]. A traction-free boundary condition was applied at the venous outlet, and zero velocity (no-slip condition) was applied at the vessel walls, which were considered to be rigid. We discretized the time-dependent terms using an implicit scheme with second-order accuracy. A total of 1000 fixed time steps were used per pulse cycle, yielding a time step size of 0.001 s and a cardiac cycle period of 1 s. For each simulation, we solved three complete cardiac cycles to avoid start-up transients, and saved only the third cycle for data processing in 100 equal time steps. A residual of 10^−5^ was set as the convergence criterion. Blood was modeled as an incompressible fluid with a constant density of 1.050 g/cm^3^, and blood viscosity was considered non-Newtonian by using the Carreau rheological model (Equation (1)):

(1) $$\mu =\mu_{\infty }+(\mu_{0}-\mu_{\infty }){\left(1+{\left(\lambda \dot{\gamma }\right)}^{2}\right)}^{\frac{n-1}{2}}$$

where $\mu_{\infty }$ is the limiting viscosity at infinite shear rate, $\mu_{0}$ is the limiting viscosity at zero shear rate, λ and n are constants, and $\dot{\gamma }$ is the strain rate [14]. The use of the Carreau model was justified by our focus on identifying venous wall regions with low WSS, for which non-Newtonian effects may be particularly significant. For the PA inlet and venous outlet, we also calculated the Reynolds and the Womersley numbers as described previously [15]. Geometric and hemodynamic features of the AVF and FCAD model 2 simulations are summarized in Supplemental Table S2. The mean and range of the Reynolds numbers predicted at the vein outlet were 1315 (1137–2228) and 1643 (1343–2382) for the AVF and FCAD, respectively, which justifies the use of a laminar flow solver.

### 2.3. Flow Field Characterization and Metric Analysis

The ability of the FCAD to normalize venous AVF flow and WSS was investigated in silico by comparing flow profiles and WSS characteristics in the idealized AVF model (i.e., reference hemodynamic state) with those in FCAD AVF models subjected to parametric changes in tab geometry. After obtaining the flow field for each AVF, we created 13 transverse cross-sections normal to the vein centerline at 6 mm increments, starting at the anastomosis. We assessed the flow profile in each cross-section using velocity contours and the following absolute and relative efficiency parameters, as previously described in the literature [10]. By comparing these parameters at multiple locations downstream of the FCAD outlet with the corresponding locations in the reference AVF model, we tailored the device performance to improve venous flow through the AVF.

We used profile symmetry, K_sym_, to assess the symmetry of the time-averaged velocity profile about the vessel centerline. This parameter represents a non-dimensional distance between the centroid of the flow profile and the vessel axis and can be calculated in any transverse cross-section of the vessel (Equation (2)):

(2) $$K_{sym}=\frac{\sqrt{x_{c}^{2}+y_{c}^{2}}}{R}$$

where R is the vessel radius, and *x*_c_ and y_c_ are the coordinates of the centroid of the mass flow given by Equation (3):

(3) $$x_{c}=\frac{\iint_{A}x\bar{v}dA}{Q}\;y_{c}=\frac{\iint_{A}y\bar{v}dA}{Q}$$

where $\bar{v}$ is the time-averaged axial velocity, Q is the volumetric flow rate, and *x* and *y* are distances from the centerline to the radial coordinates. Therefore, K_sym_ is always positive, with smaller values indicating less flow distortion.

We characterized changes in WSS through the FCAD and downstream of the venous outlet using two WSS metrics to assess regions of potential endothelial dysfunction and/or IH formation. Time-averaged WSS (TAWSS) was used to identify regions of excessively high or low WSS that occur over the cardiac cycle and is defined as:

(4) $$TAWSS=\;\frac{1}{T}\int_{0}^{T}\left|\tau_{w}\right|dt$$

where T is the period of the cardiac cycle and τ_w_ is the instantaneous WSS vector. To quantify reciprocating disturbed flow during the cardiac cycle, the oscillatory shear index (OSI, Equation (5)) was calculated on the surface of each AVF model as previously described [16]:

(5) $$OSI=\;\frac{1}{2}\left(1-\frac{\left|\int_{0}^{T}\tau_{w}dt\right|}{\int_{0}^{T}\left|\tau_{w}\right|dt}\right)$$

We also averaged these WSS parameters circumferentially within each of the 13 cross-sections, as previously described, yielding a single value for each parameter at multiple intervals along the AVF vessel [12].

On the basis of the absolute parameters presented above, we also determined the relative efficiency of the FCAD in improving each flow-field metric, ε_i_, compared to the reference AVF model, as shown in Equation (6):

(6) $$\varepsilon_{i}\left(z\right)=\frac{K_{i}^{AVF}(z)-K_{i}^{Device}(z)}{K_{i}^{AVF}(0)-K_{i}^{Device}(0)}$$

where K^AVF^ and K^Device^ represent the values of the absolute parameters calculated for the same vessel configuration without and with FCAD, respectively, and z is the distance downstream of the AVF anastomosis. These parameters quantify FCAD efficiency relative to the reference AVF as the distance downstream of the implant varies. Values of ε_i_ greater than zero show an efficiency of the FCAD greater than that of the reference AVF and allow for comparison of different FCAD geometries.

To investigate vortex generation in the wake of the flow-conditioning tabs, we also characterized the bulk flow phenotype by determining the local normalized helicity (LNH). As described by Levy et al., LNH is an effective tool for characterizing vortical structures past an axisymmetric body at a non-zero angle of attack and is expressed as [17]:

(7) $$LNH=\frac{\left(\nabla \times v\right)\cdot v}{\left|\nabla \times v\right|\cdot \left|v\right|}$$

where (∇×ν) and ν are the vorticity and the velocity vectors, respectively.

Lastly, we assessed the resistance to AVF flow imposed by FCAD implantation and the sensitivity to inlet flow. For the implant to be clinically effective, its design must impose minimal resistance to venous flow through the AVF so it does not hinder blood flow to the hemodialysis machine. Therefore, we calculated the pressure loss coefficient (resistance coefficient), ζ, for each FCAD model across a series of flow regimes with increasing Reynolds number from 760 to 2400 by scaling the PA and DA flow waveforms (see section above). Flow rates were chosen to represent flows suitable for dialysis and potentially achievable with the modeled vessel size. The pressure loss coefficient is a dimensionless quantity representing pressure loss associated with the shape of pipes and obstacles to flow and was defined as [18]:

(8) $$\zeta =\frac{P_{PA}-P_{venous\;outlet}}{0.5\rho V^{2}}$$

where V represents the mean flow velocity through the FCAD; ρ is the blood density; and P_PA_ and P_venous outlet_ are the total pressure at the PA and venous outlet, respectively. We measured pressures 2.5 mm upstream from the anastomosis in the PA and at the location corresponding to the FCAD venous outlet in all models. Compared with other measures of flow resistance, ζ captures both energy losses from viscous friction within the measurement region and the vessel’s geometric shape. For these reasons, we used it to compare FCAD resistance between each model and the idealized AVF.

### 2.4. Fabrication of AVF Flow Phantoms

A two-step, embedded scaffold removing open technology (ESCARGOT) [19] method was used to generate AVF flow phantoms to assess FCAD performance in vitro using time-resolved particle image velocimetry (PIV). Briefly, we extracted 3D models of the vessel lumens from idealized AVF models with and without the FCAD design, as described above, using SolidWorks 2015 (Dassault Systèmes, Velizy-Villacoublay, France). We converted these CAD models to STL format for 3D printing. The artificial vessels were 3D printed in acrylonitrile butadiene styrene (ABS) plastic using a commercial 3D printer (Ultimaker 2+, Ultimaker; Geldermalsen, The Netherlands) with a 0.15 mm nozzle (3D Solex, model #7072482000109) in the highest layer-resolution mode (60 μm). During post-processing, we used a small amount of acetone to smooth any rough surfaces. Each 3D ABS model was then encapsulated in transparent polydimethylsiloxane (PDMS) (Sylgard 184, Dow Corning; Midland, MI, USA) and cured under vacuum at room temperature overnight. After curing, the PDMS blocks containing the 3D ABS model were cut into a rectangular shape. Next, the blocks were placed in an acetone bath overnight to dissolve the ABS, yielding a hollow AVF flow phantom for each model. Each phantom was then rinsed with water and allowed to dry before coating the luminal surface with PEG-200 to reduce particle sticking on the channel during PIV experiments. Supplemental Figure S1A–D shows example images of this fabrication process after 3D printing, embedding, and removal of the artificial AVF vessels.

### 2.5. PIV Setup and Analysis

The AVF phantoms were incorporated into an in vitro flow system and perfused with a working fluid composed of water (55%) and glycerol (45%) to match the refractive index of transparent PDMS. Supplemental Figure S1E,F demonstrates the refractive index matching of this working fluid compared to air. Because the working fluid has higher viscosity, we scaled the flow waveforms shown in Figure 2E to match the Reynolds numbers at the PA inlet and PA outlets, respectively. A pulsatile blood pump (55-3321, Harvard Apparatus, MA, USA) propelled the working fluid into the PA of the phantom at 60 beats per minute, corresponding to a mean Womersley number of 3.4. We connected an adjustable-height afterload to the DA outlet of the phantom to achieve the desired flow ratio between the DA and vein throughout the pump cycle. The venous outlet was left free. We monitored flow rates at the PA inlet and DA outlet throughout the experiments using two perivascular flow probes (4PS and 6PS, Transonic Systems Inc., Ithaca, NY, USA) connected to a Transonic T402 flowmeter console. We also monitored pressure at the PA inlet using an inline pressure transducer (PRESS-N-025, PendoTECH; Princeton, NJ, USA). Acquisition of the flow and pressure transducer signals was performed at 1 kHz and synchronized with the PIV measurements using a data acquisition system (PowerLab, AD Instruments; Colorado Springs, CO, USA). Supplemental Figure S2 shows a schematic of the flow system setup.

Flow-velocity measurements in each phantom were obtained using time-resolved 2-D PIV. We seeded the working fluid with 2.0 µm polystyrene particles coated with Rhodamine to capture the flow field. PIV images were acquired in the mid-longitudinal plane of the vessels by illuminating the flow with a high-repetition-rate, dual-cavity Nd:YLF laser system (LDY304, Litron; Agawam, MA, USA) synchronized with a high-speed video camera (Phantom 1610, Vision Research Inc.; Wayne, NJ, USA). The laser generated a 1 mm-thick light sheet in the anastomosis region near the outflow venous segment. We chose the mid-longitudinal plane because vessel area and velocity magnitudes are typically highest in this section, and it enabled visualization of the FCAD geometry. The camera lens was fitted with a 527nm band-pass filter to minimize laser reflections. Each image captured a 60 × 40 mm area, corresponding to a pixel resolution of 1280 × 800. The PIV data was captured at 200 Hz, and each image pair was taken at a time interval of 150 μsec. Post-processing of the PIV data was performed using DAVIS 8.4 (LaVision GmbH, Goettingen, Germany) with a multi-pass, decreasing window size (64 × 64 to 32 × 32) and an adaptive interrogation window with 50% overlap. A total of 6000 image sets were acquired over 30 pump cycles and phase-averaged. We determined the phase of the velocity fields relative to the pump cycle by synchronizing the PIV measurements with the pressure transducer signal.

## 3. Results

### 3.1. Flow Patterns in the AVF and FCAD

**Figure 3:**
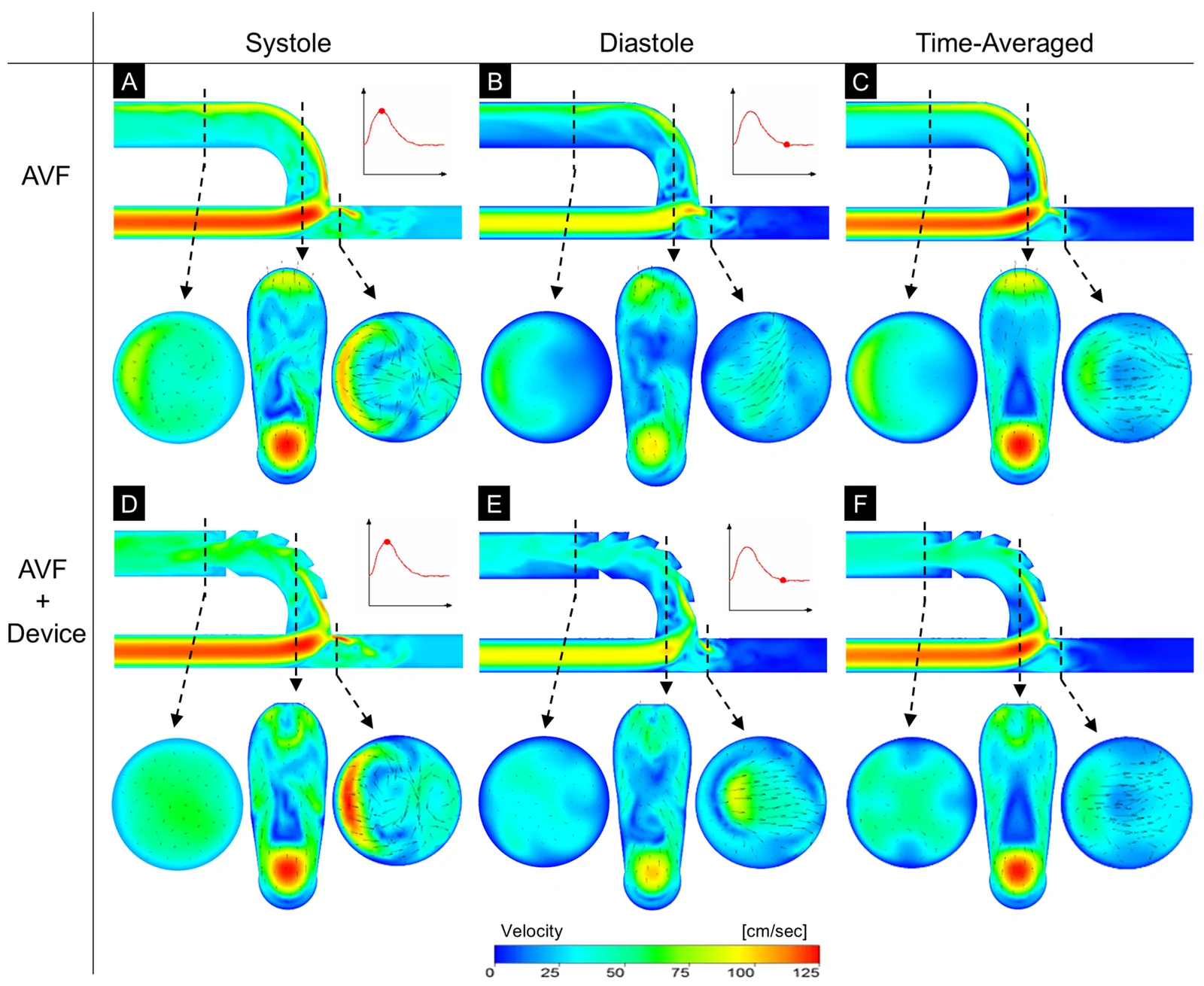
Flow field profiles in the AVF and FCAD model 2. Temporal snapshots of velocity magnitude on the longitudinal symmetry plane in idealized end-to-side AVF configurations with and without FCAD 2 implanted. Velocity contours are shown at peak systole (**A**,**D**), mid-diastole (**B**,**E**), and time-averaged across the entire cardiac cycle (**C**,**F**). Velocity vectors in three planes orthogonal to the vessel centerlines at the venous outlet, mid-anastomosis, and arterial outlet of the FCAD are indicated by dashed arrows and shown below each time-point. Solid black arrows indicate the direction of blood flow.

CFD-derived velocity contours of blood flow in the longitudinal symmetry plane of the idealized AVF and an FCAD-implanted AVF are shown in Figure 3 at peak systole, diastole, and time-averaged across the entire cardiac cycle. Representative velocity profiles orthogonal to the vessel centerlines are also shown at the venous outlet, mid-anastomosis, and arterial outlet of the FCAD in both models. In the AVF, the high-momentum flow emanating from the PA is deflected towards the outer curve of the venous swing segment, resulting in a distorted and asymmetric flow profile in the draining vein throughout the cardiac cycle (Figure 3A–C). This flow pattern creates a region of flow stagnation and recirculation along the inner curve of the venous swing segment and draining vein, which is accentuated during diastole. In addition, the sudden curvature of the AVF venous swing segment leads to the formation of Dean vortices characteristic of curved tubes. These vortices are observed in the orthogonal cross-section at the venous outlet in Figure 3A and gradually diminish along the vein.

In contrast, implantation of an FCAD with a tab angle of 30° and a height of 0.1 diameters redirects blood flow from the outer wall of the swing segment back toward the centerline of the vein (Figure 3D–F). After passing through the flow-conditioning tabs at the venous outlet of the FCAD, the region of flow stagnation along the inner curve of the draining vein is significantly reduced, and the velocity profile appears more symmetric in the longitudinal and orthogonal planes compared to the AVF. These flow characteristics persist throughout the entire cardiac cycle. Flow through the DA outlet of the FCAD is almost identical to that of the AVF due to impingement of flow on the toe of the anastomosis, resulting in a region of flow stagnation on the anastomosis floor.

### 3.2. WSS Patterns in the AVF and FCAD

**Figure 4:**
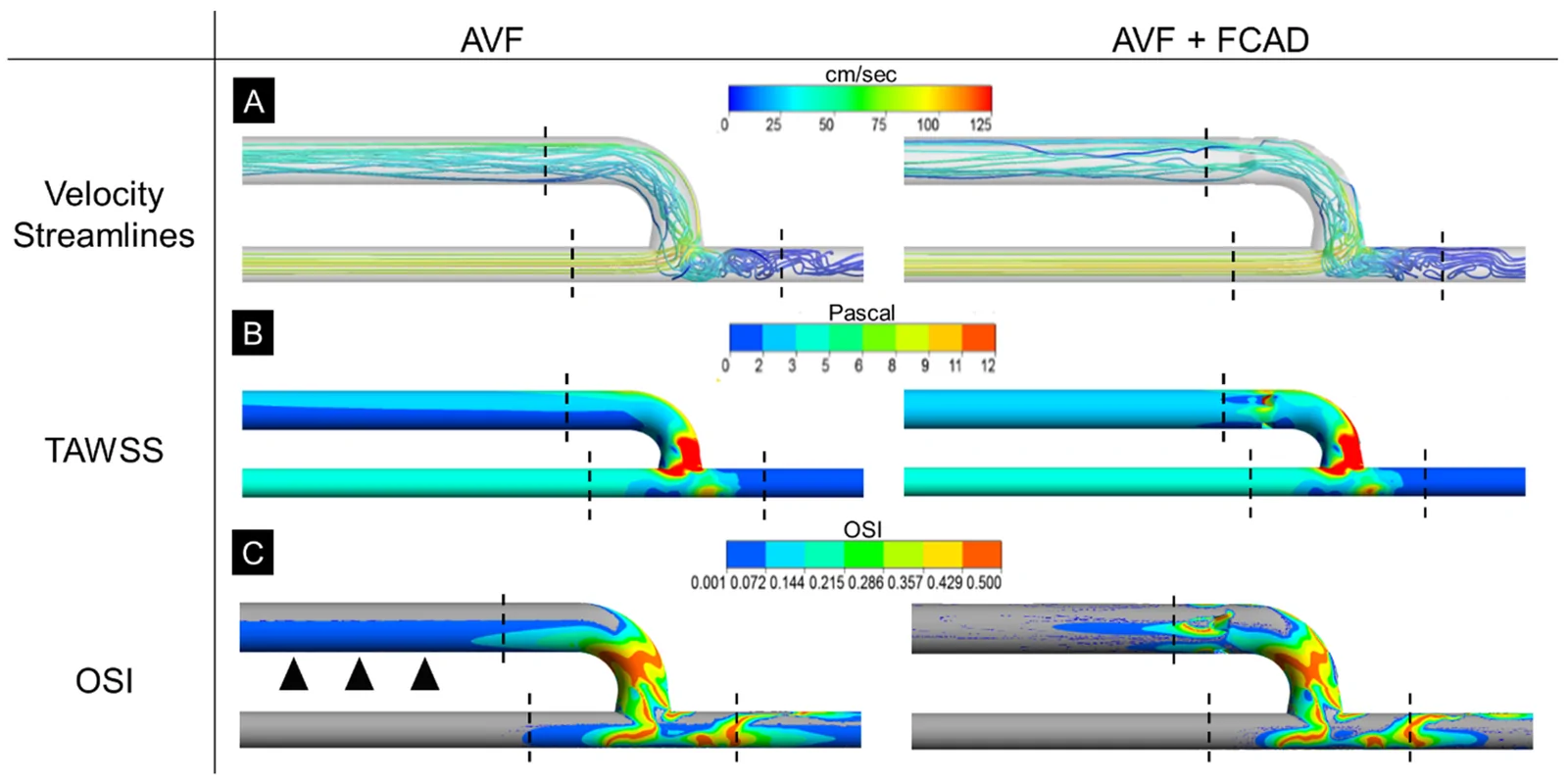
Wall shear stress analysis in AVF and FCAD model 2. (**A**) Time-averaged velocity streamlines in the reference AVF and FCAD model 2. (**B**) Time-averaged wall shear stress (TAWSS) in the reference AVF and FCAD model 2. (**C**) Oscillatory shear index (OSI) in the reference AVF and FCAD model 2. Black arrowheads highlight the differences in OSI along the inner wall of the venous outlet between the AVF and FCAD. Vertical dashed lines represent the corresponding location of the FCAD inlets and outlets in each view.

Given the relationship between regions of low, oscillatory WSS and IH formation in the venous limb of native fistulae, we assessed changes in WSS parameters in AVF models with and without the FCAD. Time-averaged flow streamlines and the corresponding TAWSS over the vessel surface in the AVF and FCAD model 2 are shown in Figure 4. The regional TAWSS distribution on the venous wall of the AVF (Figure 4B) revealed a region of locally low WSS (<1 Pa) along the inner wall of the AVF vein proximal to the anastomosis. In contrast, this region was absent in the FCAD-implanted AVF with a tab angle of 30° and a height of 0.1 diameters. However, we identified small regions of low WSS downstream of each flow-conditioning tab within the FCAD lumen. Analysis of the OSI between models demonstrated similar patterns of highly oscillatory flow (OSI > 0.4) at the anastomosis floor and the juxta-anastomotic vein (Figure 4C). In addition, small regions with OSI > 0.4 were also present around the perimeter of each flow-conditioning tab. Importantly, regions of oscillatory flow at the venous outlet were significantly smaller in the FCAD model 2 than in the reference AVF, particularly along the inner wall with low TAWSS.

### 3.3. Effect of FCAD Design on AVF WSS and Flow Field

**Figure 5:**
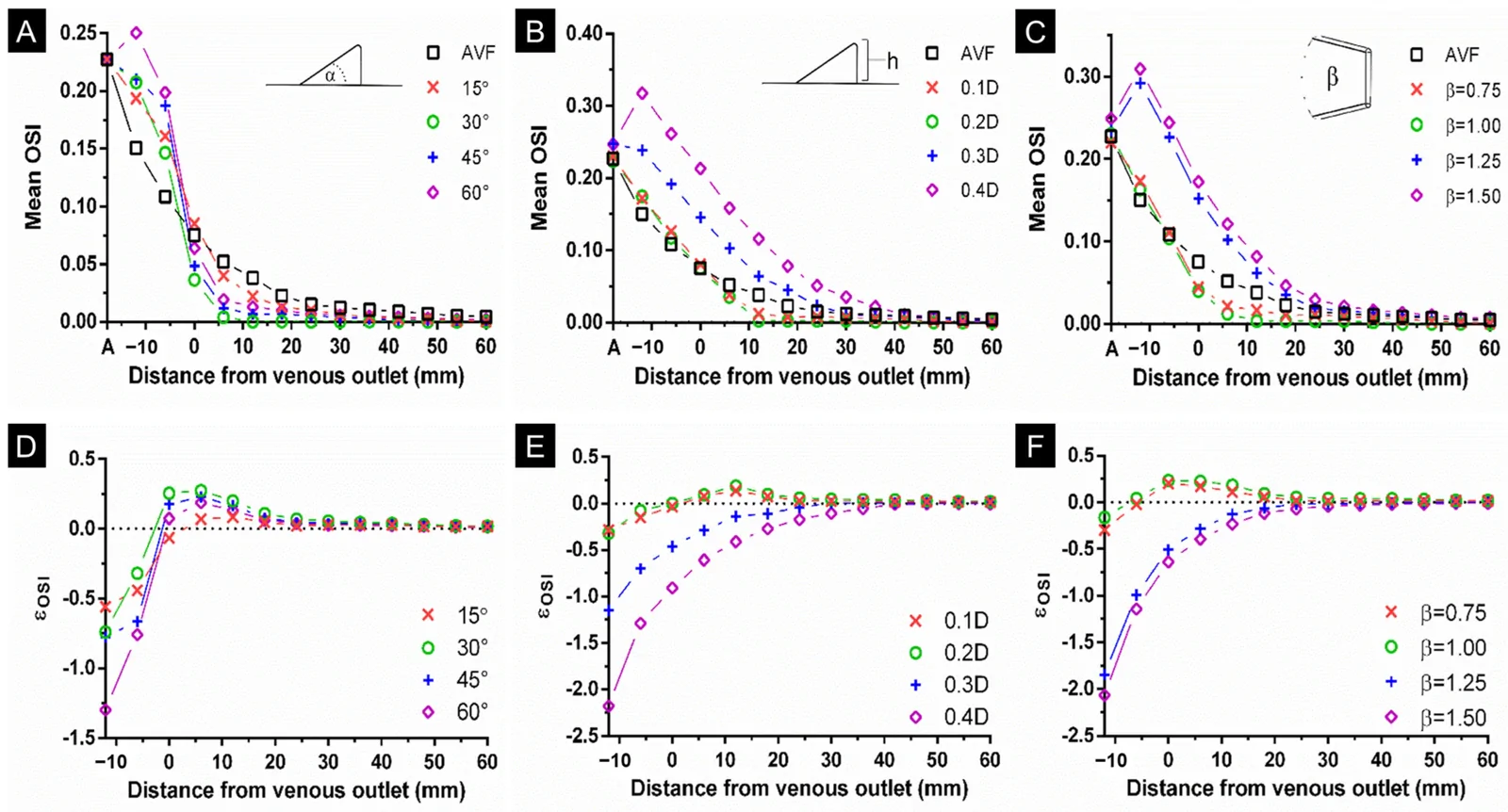
Parametric analysis of oscillatory WSS along the draining vein in AVF and FCAD models. Oscillatory shear index (OSI) was circumferentially averaged and plotted at multiple cross-sections downstream of the anastomosis in FCADs with increasing (**A**) tab angle (α), (**B**) tab height, and (**C**) tab surface area ratio. Distances are measured relative to the FCAD venous outlet, which is set to zero, with the anastomosis denoted as “A” in the axis. The equivalent axial location was used for the reference AVF, labeled “AVF”. The relative efficiency (ε) of each FCAD in improving mean OSI compared to the reference AVF was also determined for each tab angle (**D**), height (**E**), and area ratio (**F**).

To investigate the impact of tab design on flow characteristics, we circumferentially averaged OSI at multiple locations downstream of the anastomosis to compare WSS between the idealized AVF model and multiple FCAD models with parametric variations in tab angle, height, and aspect ratio. In the idealized AVF, mean OSI was highly skewed along the venous swing segment with a mean value of 0.225 and decayed exponentially downstream (labeled “AVF” in Figure 5A–C). Incorporation of an FCAD with tabs having an angle of 30–60°, a height of 0.1–0.2 diameters, or an aspect ratio of 0.75–1.00 significantly improved mean OSI downstream of the venous outlet (Figure 5A–C). In contrast, extreme tab height and aspect ratio values worsened the mean OSI downstream of the venous outlet. Interestingly, the mean OSI in the region adjacent to the FCAD implant (between the anastomosis and venous outlet) was significantly higher for most tab geometries but decayed more rapidly than the reference AVF. Calculation of the relative efficiency in improving the mean OSI, ε_OSI_, for each design compared to the reference AVF revealed that tab angles, heights, and aspect ratios of 30°, 0.1 diameters, and 1.0 resulted in the lowest mean OSI downstream of the FCAD, respectively (Figure 5D–F).

In addition, we also used the time-averaged velocity fields to compare the flow profile symmetry, K_sym_, between the idealized AVF model and each FCAD at multiple locations downstream of the venous outlet (Supplemental Figure S3). In the idealized AVF, velocity profile symmetry was highly skewed at the location corresponding to the venous outlet of the FCAD with a K_sym_ value of 0.49, and decayed exponentially downstream (labeled “AVF” in Supplemental Figure S3A–C). Incorporating an FCAD with a tab angle of 30–45°, height of 0.1–0.2 diameters, or aspect ratio of 0.75–1.25 significantly improved profile symmetry at the venous outlet. In contrast, 15° tabs had little effect on the flow profile. Calculation of the relative efficiency of the profile symmetry number, ε_Ksym_, relative to the reference AVF revealed that a tab angle, height, and aspect ratio of 30°, 0.1 diameters, and 1.0, respectively, also yielded the most symmetrical flow profile (Supplemental Figure S3D–F). Conversely, a tab height > 0.2 diameters or an aspect ratio > 1.25 produced a more distorted flow profile than the idealized AVF because of the high degree of obstruction.

### 3.4. Generation of Counter-Rotating Vortices by FCAD Tabs

**Figure 6:**
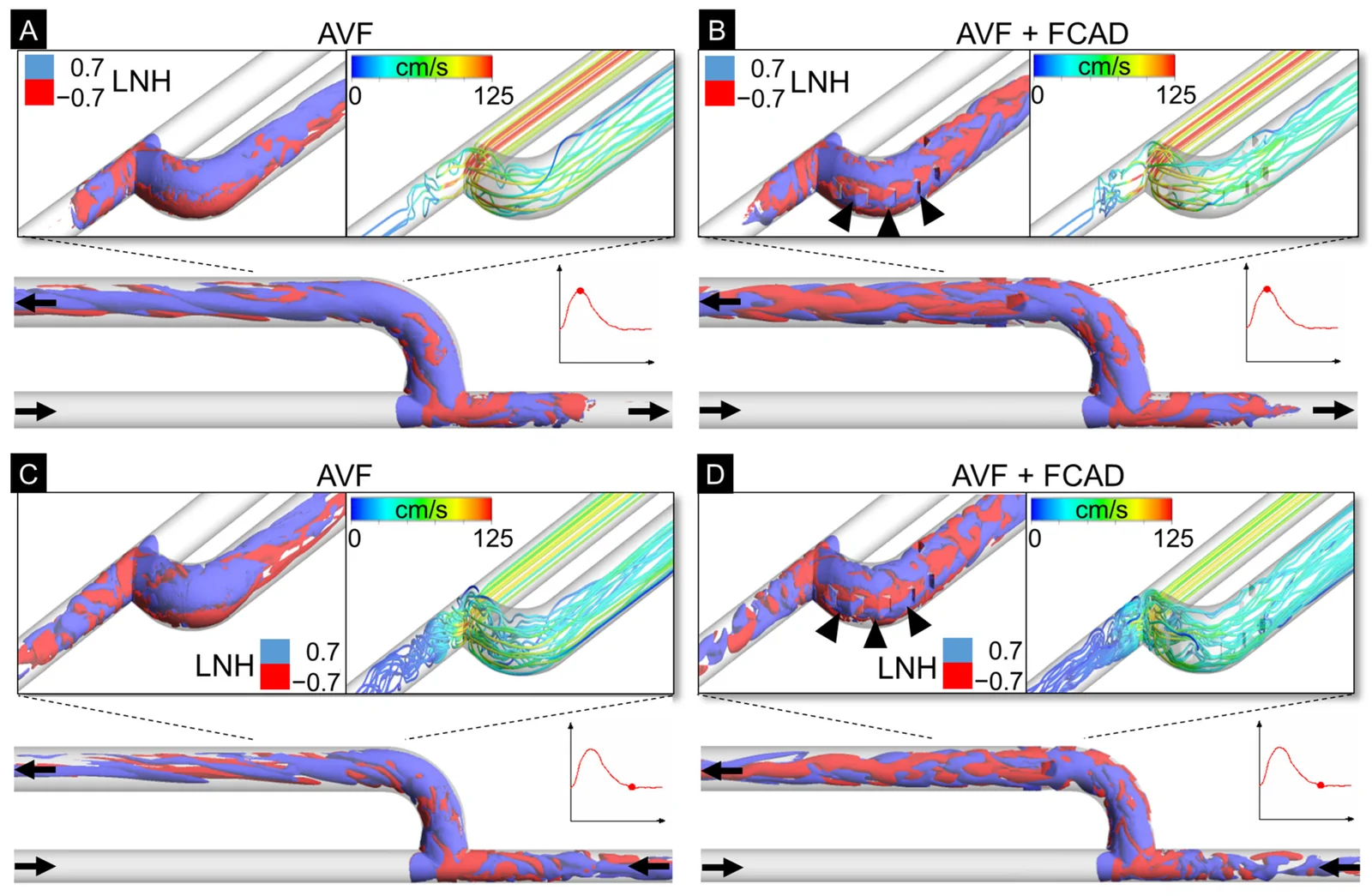
LNH isosurfaces in the AVF and FCAD model 2. Isometric and longitudinal views of local normalized helicity (LNH) isosurfaces along the venous swing segment in idealized end-to-side AVF configurations with and without FCAD 2. LNH isosurfaces are shown at peak systole (**A**,**B**) and mid-diastole (**C**,**D**). Threshold values of LNH (±0.7) are used for the identification of clockwise and counterclockwise rotating helical structures. Velocity streamlines are shown next to each isometric view for reference. Arrowheads show the generation of counter-rotating helical structures at the trailing edge of each flow-conditioning tab. Solid black arrows indicate the direction of blood flow.

Tab-type flow conditioners shed counter-rotating streamwise vortices that promote cross-stream mixing. To establish whether the FCAD generates such structures within an AVF, we computed local normalized helicity (LNH, Equation (7)), a normalized measure of the alignment between the vorticity and velocity vectors that resolves both the position and the handedness of helical flow. Figure 6 shows longitudinal and isometric views of LNH isosurfaces at peak systole and mid-diastole in AVF models with and without FCAD model 2. For reference, velocity streamlines are shown next to each isometric view. Coherent helical flow structures originate at the anastomosis in the AVF with both clockwise and counterclockwise rotation, as demonstrated by the blue and red color isosurfaces in the upper and lower surfaces of the venous swing segment, respectively (Figure 6A,C). These helical structures are present throughout the cardiac cycle, with the highest prevalence during systole. In FCAD AVF models, counter-rotating vortical structures form along the trailing edge of each tab, as shown by the reciprocal change in LNH isosurface color highlighted by black arrowheads (Figure 6B,D).

### 3.5. FCAD Imposes Minimal Resistance to AVF Blood Flow

Resistance to blood flow imposed by implantation of the FCAD was assessed by calculation of the pressure loss coefficient (Equation (8)) for each model across a series of flow regimes with increasing Reynolds number (Supplemental Figure S4). In the AVF, the pressure loss coefficient was the greatest (2.65) at the lowest Reynolds number assessed (Re = 760) and decayed exponentially with increasing Re. Incorporation of an FCAD with tabs having an angle of 15–30°, a height of 0.1–0.2 diameters, or an aspect ratio of 0.75–1.0 had minimal impact on the pressure loss coefficient, as demonstrated by the overlapping line in Supplemental Figure S4A–C. In addition, mean pressure loss across the anastomosis was similar between the AVF and FCAD model 2 at 66 (52–78) and 69 (53–81) mmHg, respectively (Supplemental Table S2). These results suggest that the FCAD geometry imposes minimal resistance to AVF flow rate. Combined with the flow-field data, tabs at 30° with a height of 0.1 diameters and an aspect ratio of 1.0 produced the most symmetric venous flow profile, with minimal oscillatory WSS and flow resistance.

### 3.6. PIV Assessment of AVF and FCAD Flow

**Figure 7:**
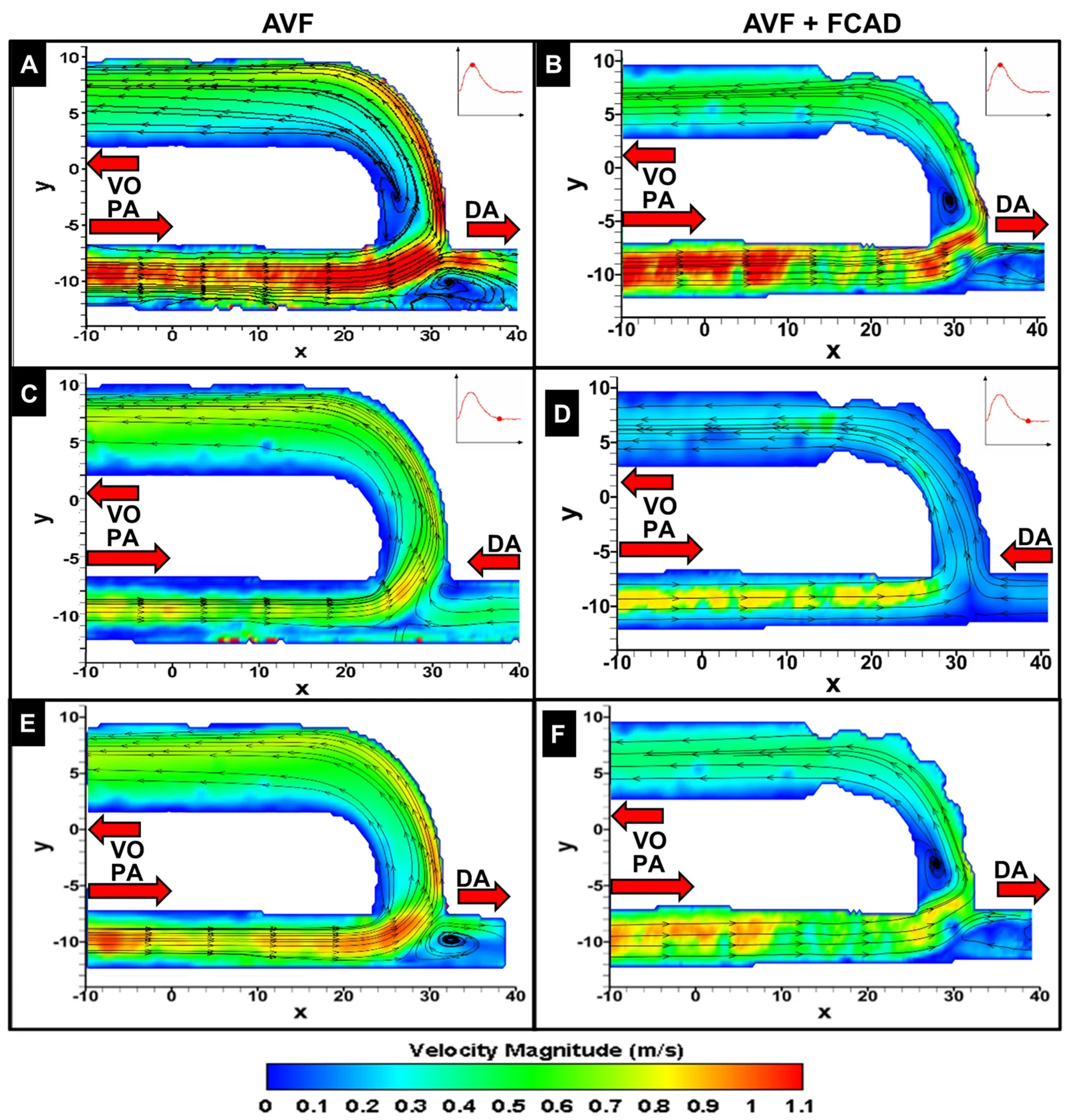
PIV-derived velocity flow fields in an idealized AVF and FCAD. Time-resolved PIV was used to quantify the flow field in the anastomotic region of AVF phantoms with and without the FCAD. Temporal snapshots of velocity magnitude on the longitudinal symmetry plane are shown at peak systole (**A**,**B**), mid-diastole (**C**,**D**), and time-averaged across the entire flow cycle (**E**,**F**). Red arrows indicate the direction of blood flow in the proximal artery (PA), distal artery (DA), and venous outlet (VO).

To validate FCAD performance in our CFD studies, we used PIV to quantify velocity flow fields in idealized AVF flow phantoms with and without the FCAD geometry. PIV-derived velocity fields in the longitudinal symmetry plane of the idealized AVF and FCAD phantoms are shown in Figure 7 at peak systole, diastole, and time-averaged across 30 flow cycles. Consistent with our CFD findings, the high-momentum flow emanating from the PA is deflected toward the outer curve of the venous swing segment in the AVF model, resulting in a distorted and asymmetric flow profile in the draining vein throughout the cardiac cycle (Figure 7A,C). In addition, flow stagnation and recirculation were observed along the inner curve of the venous swing segment and draining vein. In contrast, the FCAD phantom with a tab angle of 30° and a height of 0.1 diameters redirects flow from the outer wall of the swing segment back toward the centerline of the vein, resulting in a symmetric flow field in the downstream vein. These flow characteristics also persist throughout the entire cardiac cycle. Supplemental Figure S5 shows further similarities between the numerical CFD simulations and the PIV studies.

**Figure 8:**
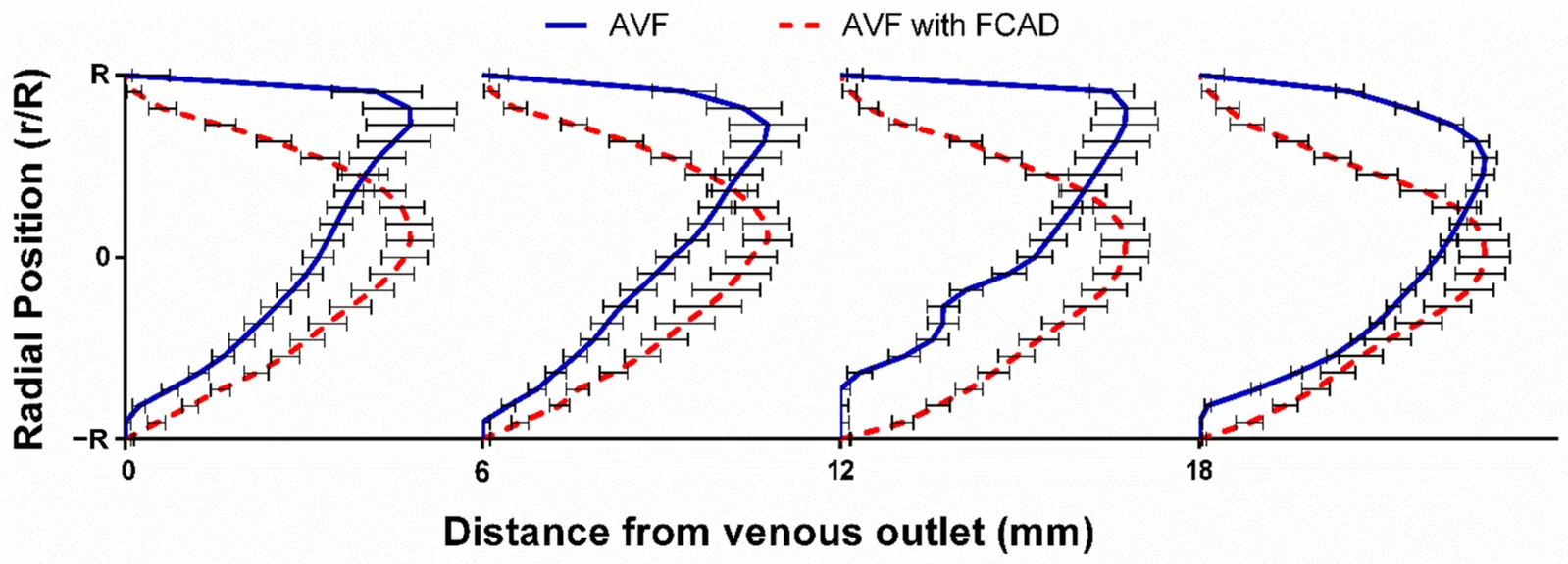
Comparison of PIV-derived axial velocity profiles in the AVF and FCAD models. Axial velocity measurements were recorded in the mid-longitudinal plane for each model and phase-averaged over 30 flow cycles. Each velocity measurement was normalized with respect to the radial position of the vessel (vessel axis = 0) and the maximum velocity. Error bars represent the root-mean-squared (RMS) error of each velocity measurement.

To further compare the flow field in the downstream vein, we quantified 2D axial velocity profiles along the mid-longitudinal plane at multiple vessel diameters downstream of the venous outlet. As shown in Figure 8, flow in the AVF model was highly skewed toward the outer wall of the venous segment, even up to 3 vessel diameters (18 mm) downstream. In contrast, the axial velocity profile in the FCAD model was highly symmetric at the venous outlet, consistent with our CFD findings. Together, these findings validate the FCAD design’s ability to improve venous flow in the hemodynamic setting of an AVF.

## 4. Discussion

Recognizing that disturbed flow affects endothelial function and vascular remodeling after AVF creation suggests that identifying an optimal AVF configuration may improve AVF maturation and patency. Several groups have investigated the influence of AVF configuration on WSS patterns and have suggested that small anastomotic angles may limit IH development [5]. Furthermore, therapeutic approaches have sought to standardize AVF configuration, either endovascularly or extravascularly, to improve AVF flow and maturation. For example, the Optiflow device was introduced to maintain a fixed anastomotic angle, and its prosthetic material acts as a shield in the juxta-anastomotic region to prevent local stenosis [20]. A clinical study of this device also confirmed its safety and surgical feasibility, with promising results [21]. Similar findings have also been reported using the VasQ external vessel support device [22]. However, as Ene-Iordache et al. concluded, it is very difficult to identify an AVF configuration that completely abolishes all areas of disturbed WSS [3]. In addition, the proximal venous segment and cephalic arch are the predominant sites of stenosis in brachiocephalic AVFs, suggesting that a greater vessel length is required for perturbations in flow to dissipate [23]. This theory is further supported by findings from McGah et al., who demonstrated only a partial restoration of homeostatic WSS 20–30 mm away from the anastomosis in mature AVFs [7]. Similarly, hemodynamic calculations estimate that a vessel length exceeding 100 diameters is required to settle swirl and asymmetric flow [18]. Therefore, we hypothesized that combining a “reasonable” configuration that shields the anastomotic region with a method to directly alter hemodynamic flow patterns in the draining vein could improve AVF outcomes.

Flow-conditioning technology is used in other applications to alter fluid flow patterns, yielding a reproducible downstream velocity profile over very short distances [24]. By combining tab-style flow-conditioning technology with a method to standardize the AVF into a curved configuration, the FCAD could restore a physiological flow profile in the draining AVF vein. Incorporating the FCAD geometry into a curved 90° AVF configuration showed that the flow-conditioning tabs restore a symmetric, physiological flow profile and TAWSS (1–3 Pa) at the venous outlet associated with AVF maturation [8]. In contrast, the reference AVF generated a large region of low TAWSS (<1 Pa) along the inner venous wall, associated with an asymmetric flow profile and increased OSI in the downstream vein. Using PIV, we also demonstrated that similar flow-field phenomena occur in AVF flow phantoms with and without the FCAD. These results also align with those of Van Canneyt et al., who observed larger zones of low WSS in AVF with a 90° anastomosis angle but decreased vortex formation [25]. A parametric analysis of tab geometry also found that tabs with an angle, height, and aspect ratio of 30°, 0.1 diameters, and 1.0, respectively, minimized regions of low and oscillatory WSS in the draining vein, characteristics shown to promote IH pathogenesis [3]. However, increasing tab height and aspect ratio were inversely related to flow-profile symmetry and yielded higher pressure losses. These findings suggest that the FCAD may effectively prevent negative vessel remodeling in the venous wall and protect the juxta-anastomotic region from low oscillatory WSS.

To be effective, a flow conditioner should have low pressure losses, compact dimensions, low cost, and reduce flow disturbances, such as asymmetry and/or swirl. These design criteria align well with therapeutic approaches to vascular access, which are hindered by high costs and poor outcomes. In particular, pressure drop is a key metric, as it inversely reflects potential flow through an AVF and indicates whether adequate blood flow can be achieved. In our parametric analyses, we demonstrate that the incorporation of flow-conditioning tabs with small tab parameters not only improves venous flow but also imposes minimal resistance to AVF flow across a series of flow regimes, as measured by the pressure loss coefficient (ζ = 2.03, 2.04, 2.03, and 2.06 for AVF and tabs with angle, height, and aspect ratio of 30°, 0.1 diameters, and 1.0, respectively, at a Reynolds number of 1390). This FACD design corresponded to a mean pressure drop of 69 mmHg, which is consistent with the computed pressure drop previously reported by Hull et al. for a 90° end-to-side AVF with the same vessel sizes and mean flow rate [26]. These computed pressure drops have also been shown to correlate well with in vivo pressure drop measurements in brachiocephalic AVFs [27].

The tab number and axial spacing were held fixed in the present study and selected based on the flow-conditioning literature, the location of flow separation predicted in the reference AVF, and constraints on hemodynamic resistance. Because tab count and spacing determine the degree to which adjacent tab wakes merge before reaching the draining vein, they are plausible design variables and may interact with tab height and angle. Screening them, together with a full-factorial or response-surface treatment of the three parameters reported here, is the subject of ongoing work.

Given the venous wall’s sensitivity to disturbed hemodynamics and injury, introducing prosthetic tabs that promote blunt-body turbulence and eddies into an autogenous AVF may be a drawback of the FCAD. However, trials of similar anastomotic devices, such as the Optiflow connector, have shown high safety after endovascular implantation to standardize the AVF configuration [21]. Nonetheless, the impact of FCAD-induced flow alterations on turbulence generation and thrombogenesis warrants investigation. Using LNH to measure bulk fluid flow, we observed a significant change in flow direction upon contact with each tab; at the trailing edges, helical vortices with opposite directions to the bulk flow were generated. These counter-rotating vortices are thought to promote cross-stream mixing that pulls fluid toward the vessel wall, enhancing the viscous boundary layer and quickly establishing a homogeneous (i.e., conditioned) flow profile [9]. Their generation is therefore the device’s intended mechanism, not an incidental effect.

Whether these associated stresses are large enough to injure the endothelium or activate platelets remains unknown. Peak instantaneous WSS on the tab surfaces and in the immediate wake reached 12 Pa at peak systole, similar to the anastomosis floor of the reference AVF and values reported in mature, clinically patent AVFs [7]. Because shear-induced platelet activation depends on accumulated stress and exposure time rather than instantaneous stress alone [28], and transit through the FCAD lumen is brief, sustained high-shear exposure appears unlikely. However, the small regions of low, oscillatory WSS around the perimeter of the tabs (Figure 4) are a concern, as thrombus nucleation on the device surface would be most plausible there. This is the central design trade-off of the FCAD. It removes a large region of low, oscillatory WSS from the native venous wall at the cost of introducing several small ones on the prosthetic surface. This trade-off cannot be fully evaluated computationally, and we are therefore pursuing in vitro thrombogenicity testing under the flow conditions modeled here alongside Lagrangian particle-tracking analysis of the CFD fields to compute stress-accumulation histories along platelet trajectories.

Although CFD has been used to evaluate pressure, flow, and WSS in idealized and patient-specific AVFs, the current study offers only a theoretical view of FCAD performance and has several limitations. First, the in silico models used to assess FCAD design and performance rely on an idealized AVF geometry with rigid walls. This assumption follows prior work on WSS and flow metrics in AVFs, and is expected to bias computed WSS upward. The magnitude of this bias depends on the fluid–structure interaction (FSI) model used and whether the geometry is initialized in a pressurized state. Studies that omit this compensation report rigid-wall overestimation of WSS near 10–13% [29]; whereas a study applying such compensation found no meaningful difference between rigid and compliant models [30]. Second, we used a single published inflow waveform and scaled its amplitude to produce the range of flow regimes reported here, but did not vary its shape. Waveform morphology depends on cardiac output, peripheral resistance, and maturation stage independently of mean flow, and the present study does not resolve that dimension of inter-patient variability. Third, although the peak-systolic Reynolds numbers calculated in the PA and venous outlet of the optimal FCAD geometry are relatively moderate (1265 and 2382, respectively), transitional and turbulent-like flow regimes with high-frequency fluctuations in streamwise velocity have been observed in the juxta-anastomotic vein [15]. Therefore, additional studies are needed to determine whether transitional turbulent effects are present in our FCAD geometries and to validate the use of a laminar flow solver. Fourth, our PIV studies were designed to validate the venous flow profile downstream of the device and do not resolve the flow immediately adjacent to each tab. Because window-correlation PIV is biased near walls and interfaces, characterizing the near-tab flow would require a magnified field of view and a near-wall method such as particle tracking velocimetry [31]. Lastly, transitional and turbulent flow regimes may also impose high shear on platelets traversing the endovascular device, leading to platelet activation and thrombosis [28]. Additional in vitro and Lagrangian approaches are being undertaken to assess platelet activation and thrombosis formation and to evaluate the risk of thrombosis in vivo [32].

In summary, this study suggests the feasibility of the FCAD to normalize flow and WSS profiles in the draining AVF vein, and identifies an ideal tab geometry with minimal resistance to AVF blood flow. Restoring physiological WSS levels on the venous wall may preserve venous endothelial function and improve AVF maturation.

## Figures and Tables

**Table 1:** Matrix of Parametric FCAD Models.

Model	Size Artery + Vein (mm)	Tab Angle (Degrees)	Height ^§^ (Diameters)	Aspect Ratio ^†^ (Width/Length)
AVF	4 + 6	-	-	-
FCAD 1	4 + 6	15	0.1	1.0
FCAD 2	4 + 6	30	0.1	1.0
FCAD 3	4 + 6	45	0.1	1.0
FCAD 4	4 + 6	60	0.1	1.0
FCAD 5	4 + 6	30	0.2	1.0
FCAD 6	4 + 6	30	0.3	1.0
FCAD 7	4 + 6	30	0.4	1.0
FCAD 8	4 + 6	30	0.1	0.75
FCAD 9	4 + 6	30	0.1	1.25
FCAD 10	4 + 6	30	0.1	1.5

§ Tab height is normalized to venous diameter. † Tab aspect ratio is defined as the tab width divided by the length. The matrix covers 11 evaluations of end-to-side AVF models with parametric changes to either the tab angle, height, or aspect ratio of the FCAD geometry. A reference AVF with an analogous configuration was used for comparison.

## Data Availability

The datasets generated and/or analyzed during the current study are not publicly available due to intellectual property considerations but are available from the corresponding author on reasonable request and with appropriate agreements in place.

## Supplementary Materials

The following supporting information can be downloaded at: https://www.mdpi.com/article/10.3390/bioengineering13091089/s1. Table S1: Discretization error of mean velocity and wall shear stress, Table S2: Parameters and flow characteristics of CFD simulations, Table S3: Constants and model parameters used for CFD simulations, Figure S1: Fabrication of AVF flow phantoms for PIV studies, Figure S2: Schematic of in vitro flow system for PIV studies, Figure S3: Parametric analysis of flow symmetry in AVF and FCAD models, Figure S4: Parametric analysis of pressure drop in AVF and FCAD models, Figure S5: Velocity field comparison between PIV and CFD studies.
