# Supplementary material for: Computational and Experimental Evaluation of a Flow-Conditioning Anastomotic Device for Arteriovenous Fistula Maturation
Source: Bioengineering (Basel). 2026 Sep 19;13(9):1089. doi: 10.3390/bioengineering13091089 (PMC13603527; doi:10.3390/bioengineering13091089)
Supplement: Supplementary file 1 [file bioengineering-13-01089-s001.zip › bioengineering-4515522-supplementary.pdf]

Supplemental Materials

Table of Contents

SUPPLEMENTAL TABLE S1: DISCRETIZATION ERROR OF MEAN VELOCITY AND WALL SHEAR STRESS........ 2

SUPPLEMENTAL TABLE S2: PARAMETERS AND FLOW CHARACTERISTICS OF CFD SIMULATIONS............... 3

SUPPLEMENTAL TABLE S3: CONSTANTS AND MODEL PARAMETERS USED FOR CFD SIMULATIONS.......... 4

SUPPLEMENTAL FIGURE S1: FABRICATION OF AVF FLOW PHANTOMS FOR PIV STUDIES 5

SUPPLEMENTAL FIGURE S2: SCHEMATIC OF *IN VITRO* FLOW SYSTEM FOR PIV STUDIES 6

SUPPLEMENTAL FIGURE S3: PARAMETRIC ANALYSIS OF FLOW SYMMETRY IN AVF AND FCAD MODELS .. 7

SUPPLEMENTAL FIGURE S4: PARAMETRIC ANALYSIS OF PRESSURE DROP IN AVF AND FCAD MODELS..... 8

SUPPLEMENTAL FIGURE S5: VELOCITY FIELD COMPARISON BETWEEN PIV AND CFD STUDIES.................. 9

Supplemental Table S1: Discretization error of mean velocity and wall shear stress

	AVF		AVF with FCAD 2	
	φ = mean velocity	φ = mean WSS	φ = mean velocity	φ = mean WSS
N_1	5,451,544	5,451,544	7,087,600	7,087,600
N_2	2,467,070	2,467,070	3,169,331	3,169,331
N_3	1,060,615	1,060,615	1,465,984	1,465,984
r_{21}	1.303	1.303	0.110	0.110
r_{32}	1.325	1.325	1.293	1.293
φ_1	44.462	37.624	45.156	43.849
φ_2	44.776	38.600	45.420	44.680
φ_3	45.913	40.721	46.020	45.942
p	4.46	2.61	3.06	1.76
φ^{21}_{ext}	44.322	36.641	44.949	42.470
e_a^{21} (%)	0.7	2.6	0.6	1.9
e_{ext}^{21} (%)	0.3	2.7	0.5	3.2
GCI^{21}_{fine} (%)	0.4	3.3	0.6	3.9

The volume-averaged mean velocity was calculated for each at the maximum venous Reynolds number during peak systole (Re = 1775). The corresponding mean WSS was also calculated for each model. AVF = arteriovenous fistula, WSS = wall shear stress, GCI grid convergence index; for the other variables see Ref. 12 of main text.

Supplemental Table S2: Parameters and flow characteristics of CFD simulations

AVF model	AVF	AVF with FCAD 2
N mesh cells	5,451,544	7,087,600
Cell vol ($x10^{-5}cm^3$)	0.37(0.0064:1.19)	0.29(0.0048:1.29)
N time steps/cycle	1,000	1,000
Delta t (s)	0.001	0.001
Q PA (mL/min)	705(563:984)	705(563:984)
Q DA (mL/min)	-22(-230:62)	-22(-230:62)
Q PA:DA:V (%)	100:23:77 – 91:9:100	100:23:77 – 91:9:100
Re PA	906(723:1265)	906(723:1265)
α PA	3.43	3.43
Re Venous Outlet	1315(1137:2228)	1643(1343:2382)
Mean Pressure Drop (mm Hg)	66(52:78)	69(53:81)

Delta t, blood flow rates, Reynolds number (Re), and pressure drop are expressed as time-averaged and (minimum:maximum) values over the pulse cycle. Q PA:DA:V represents the blood flow division ratio between these limbs of the AVF throughout the cardiac cycle. Cell vol represents the volume of the cells of the mesh expressed as mean(minimum:maximum). Q, volumetric blood flow rate; PA, proximal artery; DA, distal artery; V, vein; Re, Reynolds number; α, Womersley number.

Supplemental Table S3: Constants and model parameters used for CFD simulations

Parameter	Symbol	Value
Blood density	ρ	1050 kg/m³
Zero-shear-rate viscosity	μ_0	0.056 Pa·s
Infinite-shear-rate viscosity	μ_∞	0.0035 Pa·s
Carreau time constant	λ	3.313 s
Carreau power-law index	n	0.3568
Arterial diameter	D_a	4.0 mm
Venous diameter	D_v	6.0 mm
Cardiac cycle period	T	1.0 s
Cycles simulated / retained*	—	3 / 1
Boundary layer cells / growth rate	—	5 / 1.2

* additional mesh and time-stepping parameters are given in Supplemental Table 2

Supplemental Figure S1: Fabrication of AVF flow phantoms for PIV studies

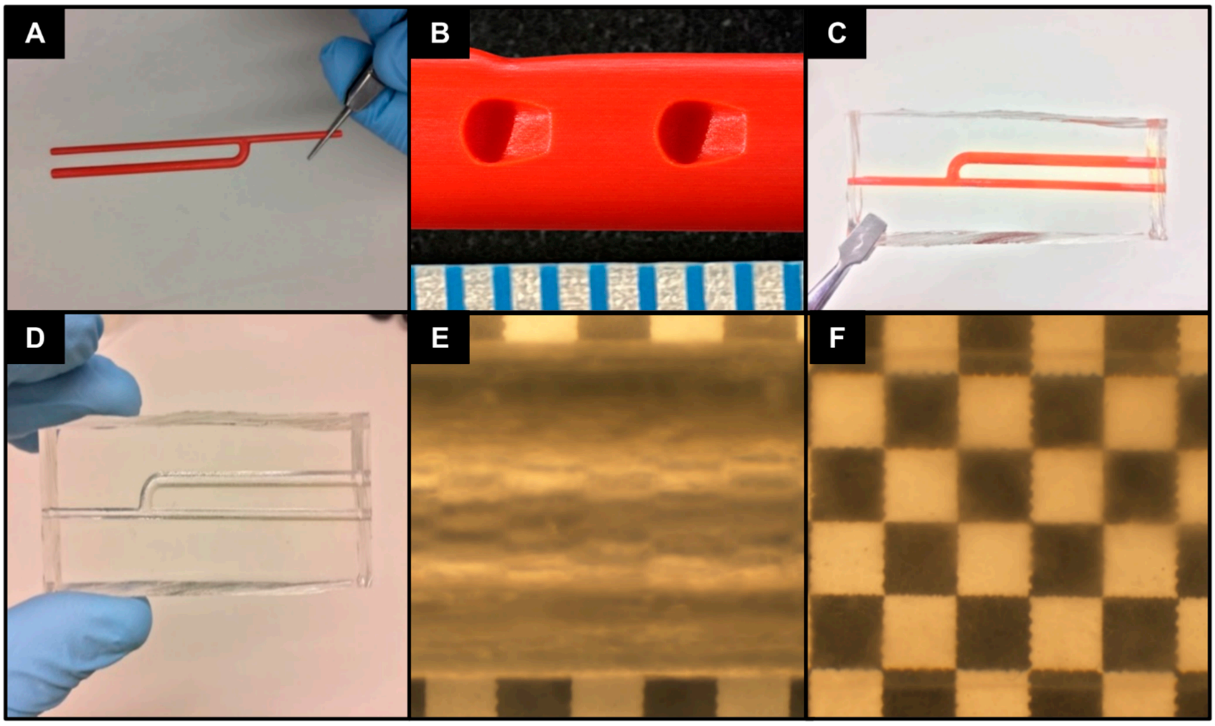

Supplemental Figure S1: Fabrication of AVF flow phantoms for PIV studies. [A] Example of 3D-printed AVF model at a 4:1 scale. **[B]** Magnified view of the flow-conditioning tabs along the outer curve of the venous swing segment on the printed positive model. Scale bar in mm. **[C-D]** Pictures of the fabrication process after embedding the model in PDMS (C) and subsequently dissolving the model to produce a hollow phantom (D). **[E-F]** Refractive index matching tests for an AVF phantom filled with (E) air, and (F) a refractive index-matched working fluid of glycerol and water. Note the absence of refraction in the background tile pattern with the index-matched fluid compared to air. AVF, arteriovenous fistula; PDMS, polydimethylsiloxane; PIV, particle imaging velocimetry.

Supplemental Figure S2: Schematic of *in vitro* flow system for PIV studies

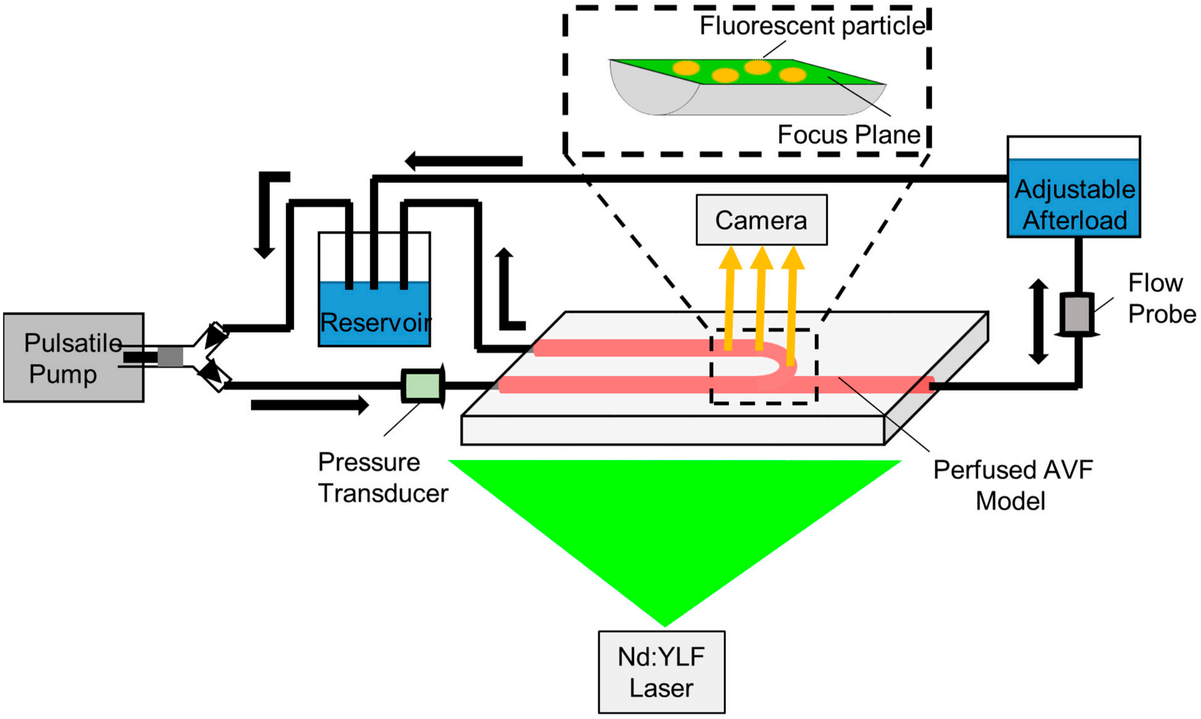

Supplemental Figure S2: Schematic of in vitro flow system for PIV studies. A working fluid containing fluorescent particles was perfused with a pulsatile blood pump through the AVF flow phantoms. PIV imaging was performed at the anastomosis and venous outlet using a Nd:YFL laser to excite the particles, and a high-speed camera to record particle motion.

Supplemental Figure S3: Parametric analysis of flow symmetry in AVF and FCAD models

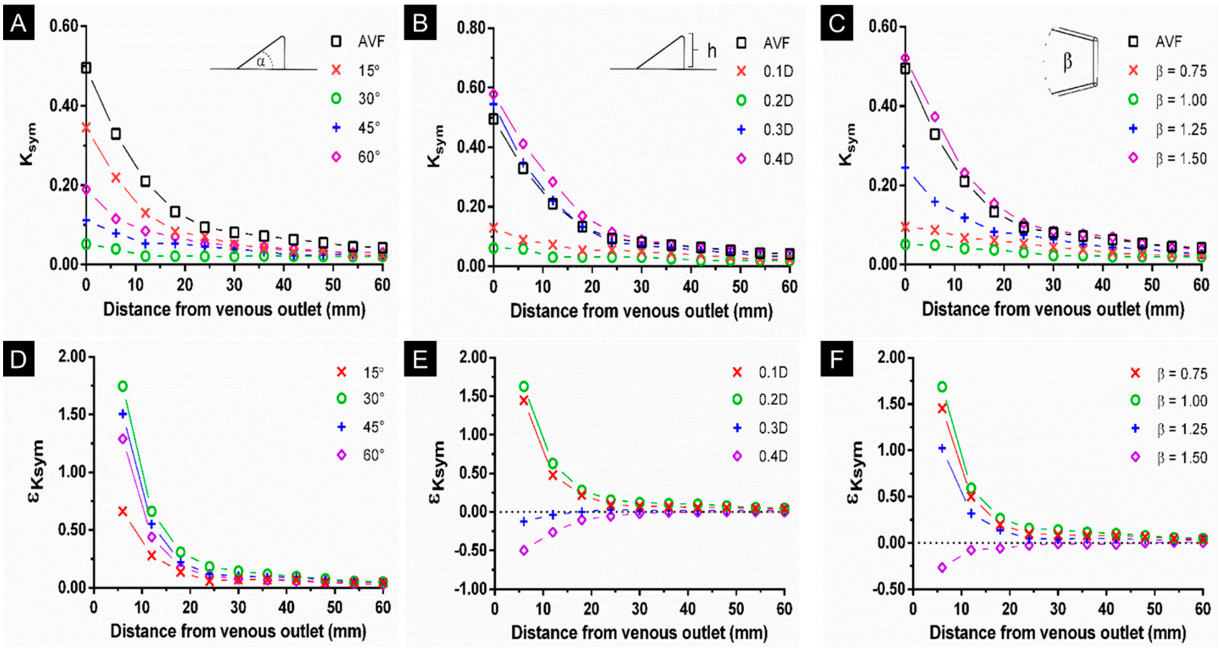

Supplemental Figure S3: Parametric analysis of flow field symmetry along the draining vein in AVF and FCAD models. Symmetry number, Ksym, was calculated at multiple cross-sections downstream of the anastomosis in FCADs with increasing [**A**] tab angle (α), [**B**] tab height, and [**C**] tab surface area ratio. Distances are measured relative to the FCAD venous outlet, which is set to zero; the equivalent axial location was used for the reference AVF, labeled “AVF”. The relative efficiency (ε) of each FCAD at improving the symmetry number, εKsym, compared to the reference AVF was also determined for each tab angle (**D**), height (**E**), and area ratio (**F**).

Supplemental Figure S4: Parametric analysis of pressure drop in AVF and FCAD models

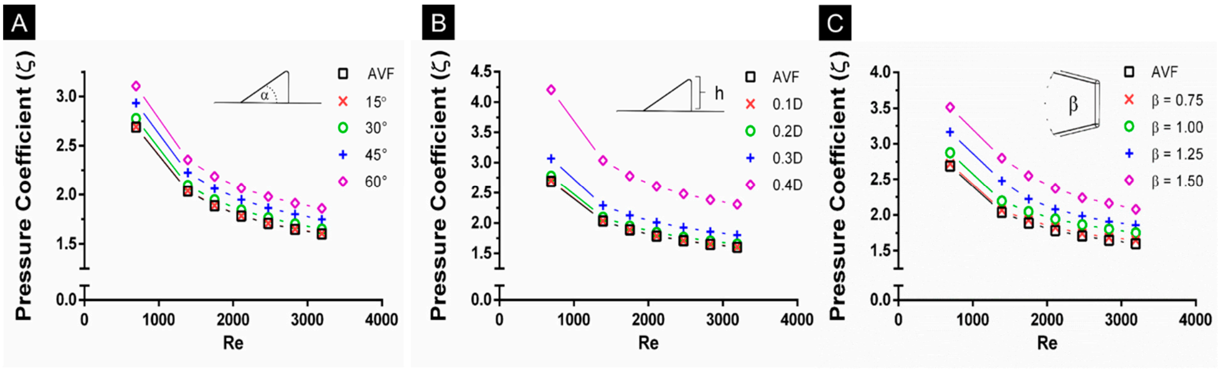

Supplemental Figure S4: Parametric analysis of pressure drop in AVF and FCAD models. The pressure loss coefficient, ζ, was calculated for FCADs with increasing [**A**] tab angle, [**B**] tab height, and [**C**] tab surface area ratio across a series of flow regimes with increasing Reynolds number (Re). The reference AVF without an FCAD is also shown.

Supplemental Figure S5: Velocity field comparison between PIV and CFD studies

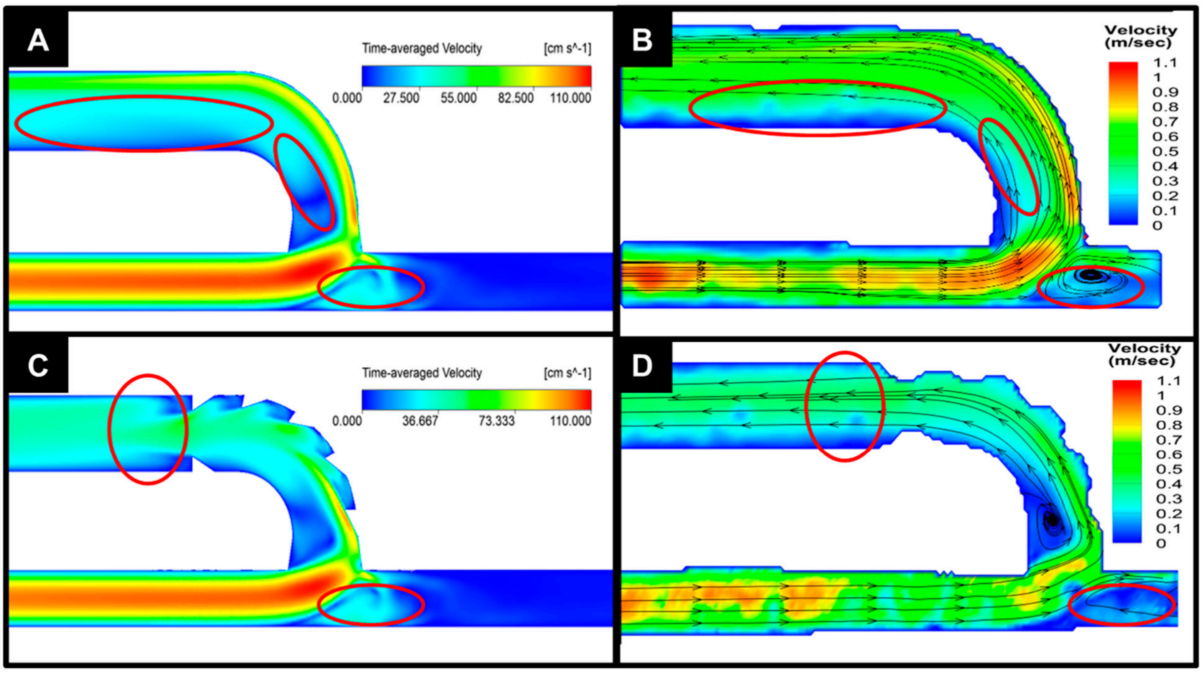

Supplemental Figure S5: Velocity field comparison between the PIV experimental results and the CFD simulations. The circled red regions show similar time-averaged flow features between the CFD and PIV results in both the AVF model [**A & B**] and FCAD [**C & D**] models. All pictures are on the same color scale shown in the CFD images.
